# Association between iron status and thyroid function in Nepalese children

**DOI:** 10.1186/s13044-016-0031-0

**Published:** 2016-01-27

**Authors:** Saroj Khatiwada, Basanta Gelal, Nirmal Baral, Madhab Lamsal

**Affiliations:** Department of Biochemistry, Modern Technical College, Lalitpur, Nepal; Department of Biochemistry, B P Koirala Institute of Health Sciences, Ghopa, Dharan, Nepal

**Keywords:** Anemia, Iron deficiency, Nepal, School children, Thyroid dysfunction, Thyroid hormone

## Abstract

**Background:**

Deficiencies of iodine and iron may have adverse effect on thyroid function. This study was undertaken to investigate the association between iron status and thyroid function in Nepalese children living in hilly regions.

**Methods:**

A cross-sectional study was conducted among 227 school children aged 6–12 years living in hilly regions of eastern Nepal. Urine and blood samples were analyzed for urinary iodine concentration, free thyroxine, free triiodothyronine, thyroid stimulating hormone, hemoglobin, serum iron and total iron binding capacity, and percentage transferrin saturation was calculated.

**Results:**

The cohort comprised euthyroid (80.6 %, *n* = 183), overt hypothyroid (1.3 %, *n* = 3), subclinical hypothyroid (16.3 %, *n* = 37) and subclinical hyperthyroid (1.8 %, *n* = 4) children respectively. About 35.2 % (*n* = 80) children were anemic, 43.6 % (*n* = 99) were iron deficient and 19.8 % (*n* = 45) had urinary iodine excretion < 100 μg/L. Hypothyroidism (overt and subclinical) was common in anemic and iron deficient children. The relative risk of having hypothyroidism (overt and subclinical) in anemic and iron deficient children was 5.513 (95 % CI: 2.844−10.685, *p* < 0.001) and 1.939 (95 % CI: 1.091-3.449, *p* = 0.023) respectively as compared to non-anemic and iron sufficient children. Thyroid stimulating hormone had significant negative correlation with hemoglobin (*r* = −0.337, *p* < 0.001) and transferrin saturation (*r* = −0.204, *p* = 0.002).

**Conclusions:**

Thyroid dysfunction, iron deficiency and anemia are common among Nepalese children. In this cohort, anemic and iron deficient children had poor thyroid function.

## Background

Multiple micronutrient deficiencies are still a major public health problem faced by developing countries. Such deficiencies have adverse effects on growth and development, especially in vulnerable groups like pregnant women and children [1]. Deficiencies of trace elements like iodine, iron, zinc and selenium also impairs thyroid function [2]. Thyroid hormones influence a number of metabolic pathways and physiological components. Excess thyroid hormones cause hyperthyroidism whereas deficiency causes hypothyroidism [3]. Iodine deficiency and iron deficiency are considered as the most common cause of preventable brain damage and anemia respectively in developing countries like Nepal [4, 5]. Anemia and iron deficiency have been found to be highly prevalent among Nepalese school children, whereas iodine nutrition has been continuously improving in school children [6, 7]. Iron deficiency with or without anemia affects child development and also has multiple adverse effects on thyroid metabolism [8, 9].

Studies have reported the association of thyroid function with iron status [10–12]. In the study by Metwalley et al. in Egypt, it was found that primary school children with iron deficiency anemia were liable to develop subclinical hypothyroidism and intellectual dysfunction [12]. Since, iron deficiency and anemia are highly prevalent in the Nepalese population, assessment of thyroid function in such a population may help to determine the potential effects of iron deficiency on thyroid metabolism [6]. Thyroid dysfunction has been also been reported more frequently in Nepalese population although iodine nutrition has been improving continuously [13, 14]. It is however, unknown about the factors responsible for thyroid disorders in this region. It has been hypothesized that iron deficiency negatively affects thyroid function leading to increased rate of thyroid disorders [10]. Thus, there might be potential link between iron deficiency and thyroid dysfunction in this population as well. Taking in to account of poor iron status and higher rates of thyroid dysfunction in Nepalese population, we tried to find the possible effects of iron deficiency on thyroid function in Nepalese children by conducting the present study.

Specifically, we selected school children aged 6–12 years randomly from hilly regions of eastern Nepal and analyzed urine and blood samples to assess iodine status, iron status and thyroid function, and determine their association.

## Methods

A cross-sectional study was conducted among children of two hilly districts (Sankhuwasabha and Dhankuta) of eastern Nepal in 2014. We assessed iodine status, iron status and thyroid function to find the association between iron status and thyroid function in the cross sectional population. Six schools (three schools in each district) were picked randomly and school children aged 6–12 years whose parents gave consent were enrolled randomly in the study. A total of 227 children (124 from Sankhuwasabha and 103 from Dhankuta district) were selected for the study. The ethical clearance for this study was given by the Institutional Review Board of B P Koirala Institute of Health Sciences (BPKIHS), Dharan. Sample size estimation was done on the basis of approximate prevalence of iodine deficiency (15 %), iron deficiency (30 %) and thyroid dysfunction (20 %) in Nepalese children.

Demographic variables (age, gender), urine samples (10 ml) and venous blood samples (3 ml) were collected from each child. The samples were transported to biochemistry laboratory of B P Koirala Institute of Health Sciences under refrigeration. Hemoglobin (Hb) was measured within 24 h of sample collection by cyanmethemoglobin method, whereas other parameters were estimated within a week of sample collection [15]. Urinary iodine excretion (UIE) was measured by using ammonium persulphate digestion method (APDM) [16]. Serum iron and total iron binding capacity (TIBC) were measured by colorimetric methods using commercial kits (Roche Diagnostics). Transferrin saturation was calculated using serum iron and TIBC value [17]. Thyroid hormones (free triiodothyronine (fT3), free thyroxine (fT4) and thyroid stimulating hormone (TSH)) were measured by immunoassay methods using ELISA kits from HUMAN Diagnostics. Normal reference range provided by kits for serum iron was 37–145 μg/dl for women and 59–158 μg/dl for men and 274–385 μg/dl for TIBC. Similarly the normal reference range for thyroid hormones were fT3 (1.4–4.2 pg/ml), fT4 (0.8-2.2 ng/dl) and TSH (0.39-6.16 mIU/L) according to kit provider.

The data generated from the study were entered in ms-excel and analyzed using SPSS version 19 software. Continuous variables were expressed as mean ± SD, except for UIE and TSH (expressed as median) and categorical variables as number (percentage). Independent *t* test, one way ANOVA, Man whitney test and Kruskal Wallis test was applied for continuous variables and chi-square test for categorical variables at 95 % confidence interval. Pearson correlation and Spearman’s rho correlation analysis was done among variables to see the association between iron status indicator and thyroid hormones at 95 % confidence interval. Relative risk for hypothyroidism in anemic and iron deficient as compared to non-anemic and iron sufficient was calculated at 95 % confidence interval.

## Results

Among the study population, 55.5 % (*n* = 126) were males and 44.5 % (*n* = 101) were females. The median UIE and TSH with IQR was 222 μg/L (121 μg/L; 296 μg/L) and 4.0 mIU/L (2 mIU/L; 6 mIU/L) respectively. The mean level of Hb, serum iron, TIBC, transferrin saturation, fT3, fT4 and TSH were 12.2 ± 1.9 gm/dl, 70.5 ± 35.4 μg/dl, 382.8 ± 89.5 μg/dl, 20.1 ± 12.6 %, 2.7 ± 0.8 pg/ml, 1.2 ± 0.4 ng/dl and 4.4 ± 2.8 mIU/L respectively in the study population, which are all in normal range. No significant differences in UIE (*p* = 0.98), Hb (*p* = 0.078), serum iron (*p* = 0.594), TIBC (*p* = 0.781), transferrin saturation (*p* = 0.766), fT3 (*p* = 0.912), fT4 (*p* = 0.842) and TSH (*p* = 0.383) were observed among gender. On the basis of Hb cutoff value for anemia for different age group and transferrin saturation cutoff < 16 for iron deficiency, 35.2 % (*n* = 80) and 43.6 % (*n* = 99) children were anemic and iron deficient respectively [18]. About, 19.8 % (*n* = 45) children had UIE < 100 μg/L, which is considered as iodine deficiency on the basis of WHO criteria for iodine status [19]. Level of biochemical parameters according to anemia and iron status is shown in Table 1. Median TSH level was higher in anemic than non-anemic (*p* < 0.001), but similar in iron deficient and iron sufficient (*p* = 0.05). fT3 was significantly higher in iron sufficient than iron deficient (*p* = 0.003). Level of Hb, serum iron, TIBC and transferrin saturation was 12.3 ± 2.1 gm/dl versus 12.3 ± 1.9 gm/dl (*p* = 0.924), 81.4 ± 35.9 μg/dl versus 67.9 ± 34.9 μg/dl (*p* = 0.022), 371.7 ± 97.5 μg/dl versus 385.6 ± 87.6 μg/dl (*p* = 0.354), and 23.8 ± 12.8 % versus 19.3 ± 12.5 % (*p* = 0.032) among iodine deficient and iodine sufficient children respectively. Similarly, the mean level of fT3, fT4 and median TSH was 2.8 ± 0.9 pg/ml versus 2.7 ± 0.8 pg/ml (*p* = 0.78); 1.2 ± 0.5 ng/dl versus 1.2 ± 0.4 ng/dl (*p* = 0.567) and 4.0 mIU/L (2; 6) versus 4.0 mIU/L (2; 6) (*p* = 0.953) among iodine deficient and iodine sufficient respectively.

**Table 1:** Biochemical parameters in study population according to anemia and iron status

Variables	Total	Anemia status			Iron status		
		Anemic	Non-anemic	*P* value	Iron deficient	Iron sufficient	*P* value
	*N* = 227	*N* = 80	*N* = 147		*N* = 99	*N* = 128	
UIE (μg/L)	222 (121; 296)	225 (112.7; 303.5)	222 (125; 281)	0.788	228 (133; 307)	220 (119.5; 291)	0.428
Hemoglobin (gm/dl)	12.2 ± 1.9	10.3 ± 1.0	13.3 ± 1.3	<0.001	11.6 ± 1.8	12.7 ± 1.7	<0.001
Serum iron (μg/dl)	70.5 ± 35.4	55.4 ± 21.8	78.8 ± 38.6	<0.001	39.6 ± 12.9	94.4 ± 28.1	<0.001
TIBC (μg/dl)	382.8 ± 89.5	414.6 ± 81.5	365.4 ± 89.2	<0.001	433.2 ± 81.9	343.8 ± 74.8	<0.001
Transferrin saturation (%)	20.1 ± 12.6	14.2 ± 7.6	23.4 ± 13.6	<0.001	9.2 ± 2.5	28.6 ± 10.7	<0.001
Free T3 (pg/ml)	2.7 ± 0.8	2.6 ± 0.9	2.8 ± 0.7	0.13	2.6 ± 0.8	2.9 ± 0.7	0.003
Free T4 (ng/dl)	1.2 ± 0.4	1.2 ± 0.5	1.2 ± 0.4	0.526	1.2 ± 0.5	1.2 ± 0.4	0.531
TSH (mIU/L)	4.0 (2; 6)	5.0 (3; 8)	3.0 (2; 5)	<0.001	4.0 (3; 7)	4.0 (2; 6)	0.05

The data is expressed as mean ± SD except for UIE and TSH (expressed as median with IQR). *P* value was calculated at 95 % confidence interval

Among the study children, 80.6 % (*n* = 183), 1.3 % (*n* = 3), 16.3 % (*n* = 37) and 1.8 % (*n* = 4) were euthyroid, overt hypothyroid, subclinical hypothyroid and subclinical hyperthyroid respectively. Thyroid status according to anemia and iron deficiency is shown in Table 2. Similarly, level of UIE, Hb and iron status indicators in the study population according to thyroid function status is shown in Table 3. No significant difference in number of euthyroid (36 versus 147, *p* = 1.0), overt hypothyroid (2 versus 1, *p* = 0.101), subclinical hypothyroid (7 versus 30, *p* = 1.0) and subclinical hyperthyroid (0 versus 4, *p* = 0.587) was observed among iodine deficient and iodine sufficient children.

**Table 2:** Thyroid function status in study population according to anemia and iron status

Variables	Total	Anemia status			Iron status		
		Anemic	Non-anemic	*P* value	Iron deficient	Iron sufficient	*P* value
	*N* = 227	*N* = 80	*N* = 147		*N* = 99	*N* = 128	
Euthyroidism, n (%)	183 (80.6 %)	49 (21.6 %)	134 (59 %)	<0.001	72 (31.7 %)	111 (48.9 %)	0.011
Overt hypothyroidism, n (%)	3 (1.3 %)	3 (1.3 %)	-	0.043	3 (1.3 %)	-	0.082
Subclinical hypothyroidism, n (%)	37 (16.3 %)	27 (11.9 %)	10 (4.4 %)	<0.001	21 (9.3 %)	16 (7 %)	0.102
Subclinical hyperthyroidism, n (%)	4 (1.8 %)	1 (0.4 %)	3 (1.3 %)	1.0	3 (1.3 %)	1 (0.4 %)	0.32

The data is expressed as number (percentage). *P* value was calculated at 95 % confidence interval

**Table 3:** Biochemical parameters in study population according to thyroid function

Variables	Euthyroid	Hypothyroid*	Subclinical hyperthyroid	*P* value
	*N* = 183	*N* = 40	*N* = 4	
UIE (μg/L)	213 (120; 290)	236.5 (155.5; 301.5)	219 (141.7; 295.5)	0.345
Hemoglobin (gm/dl)	12.6 ± 1.7	10.7 ± 2.0	13.9 ± 2.5	<0.001
Serum iron (μg/dl)	74.0 ± 36.7	57.0 ± 26.6	50.5 ± 23.3	0.012
TIBC (μg/dl)	374.7 ± 84.2	418.8 ± 104.8	393.4 ± 101.1	0.018
Transferrin saturation (%)	21.5 ± 13.2	14.6 ± 8.1	14.0 ± 8.7	0.004

The data is expressed as mean ± SD except for UIE (expressed as median with IQR). Asterik (*) indicates that hypothyroid group included both overt and subclinical hypothyroid. *P* value was calculated at 95 % confidence interval

**Fig. 1:**
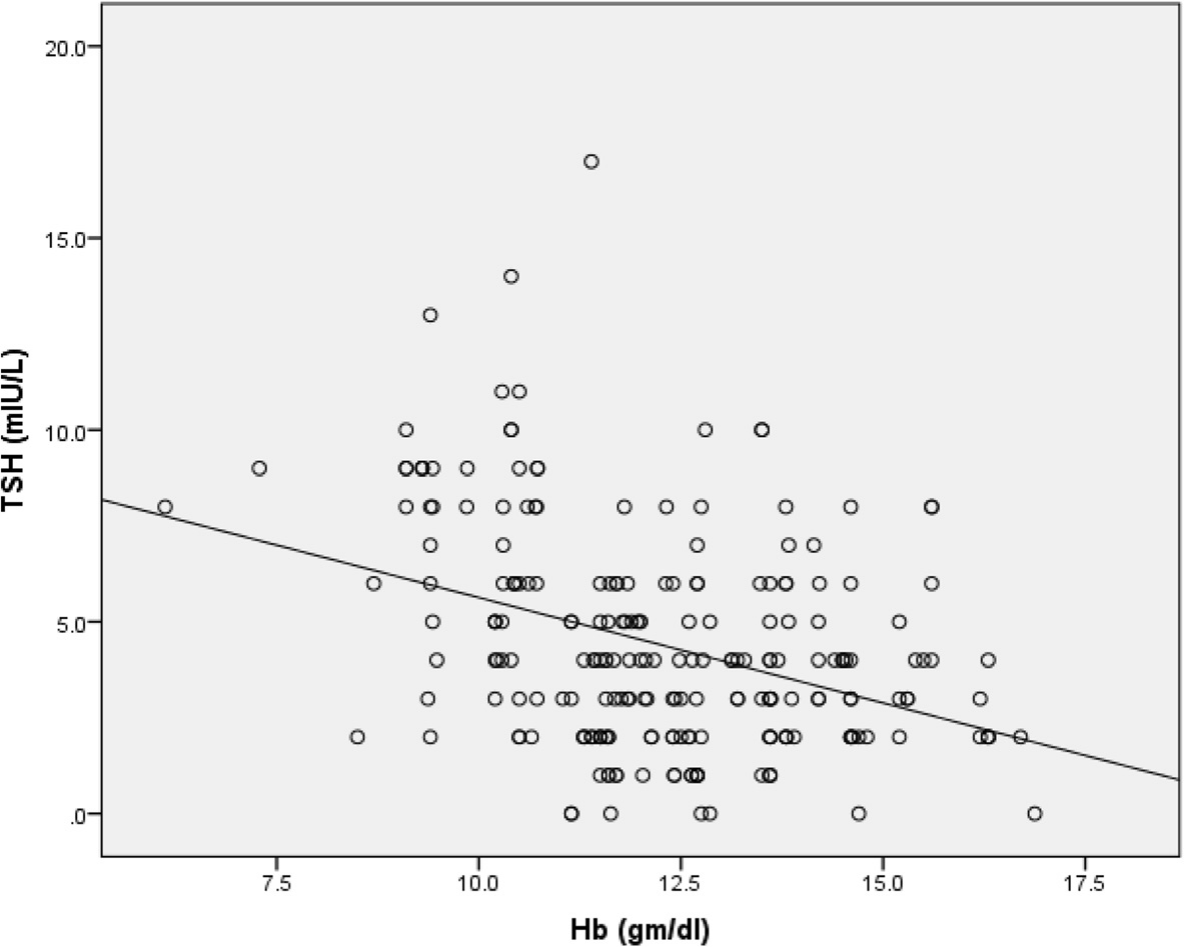
Correlation between TSH level and Hb level

**Fig. 2:**
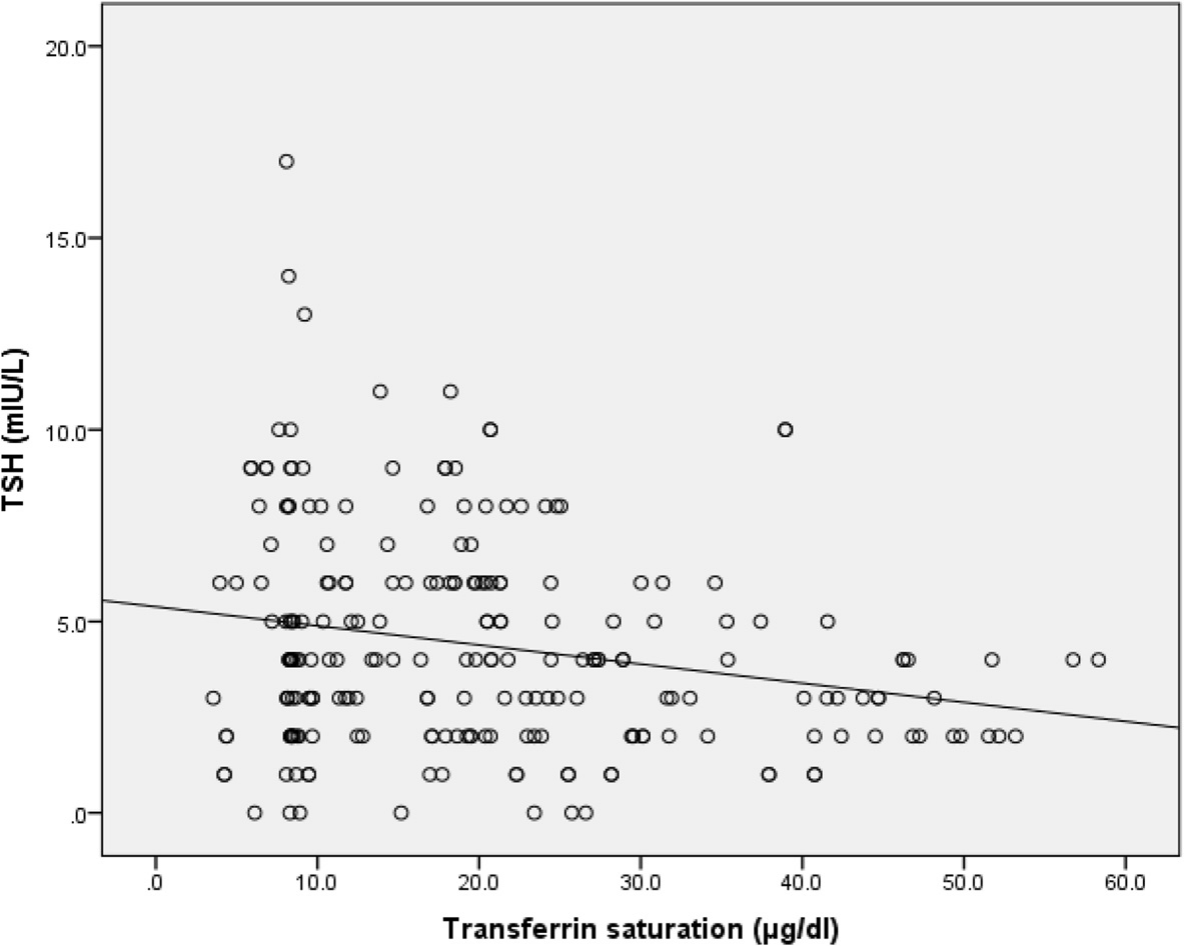
Correlation between TSH level and transferrin saturation

A significant positive correlation was observed between Hb level and transferrin saturation (*r* = 0.411, *p* = <0.001). Correlation of Hb and transferrin saturation with TSH level is shown in Figs. 1 and 2 respectively. Transferrin saturation was significantly negatively correlated with TSH (*r* = −0.204, *p* = 0.002), positively with fT3 (*r* = 0.13, *p* = 0.051) and weakly negatively with fT4 (*r* = −0.089, *p* = 0.182). Similarly, Hb was significantly negatively correlated with TSH (*r* = −0.337, *p* < 0.001), but weakly correlated with fT3 (*r* = 0.077, *p* = 0.249) and fT4 (*r* = −0.018, *p* = 0.787). UIE was very weakly correlated with TSH (*r* = 0.031, *p* = 0.643), fT3 (*r* = −0.014, *p* = 0.832) and fT4 (*r* = 0.047, *p* = 0.484). The risk of having hypothyroidism (overt and subclinical) in anemic and iron deficient children was 5.513 (95 % CI: 2.844-10.685, *p* < 0.001) and 1.939 (95 % CI: 1.091-3.449, *p* = 0.023) respectively as compared to non-anemic and iron sufficient children.

## Discussion

Iodine and iron deficiencies are very common in the regions where adequate iodine and iron is not present in normal diet [20]. We observed anemia and iron deficiency in 35.2 % and 43.6 % children respectively. Anemia and iron deficiency have been found highly prevalent in Nepalese school children. Study by Khatiwada et al. reported anemia and iron deficiency in 34.5 % and 43.4 % school children respectively [6]. The anemia prevalence according to Nepal demographic and health survey, Baral et al. study and Khatiwada et al. study were 48 % (among age 6–59 months), 65.6 % (among age 10–19 years) and 37.9 % (among age 4–13 years) respectively [7]. Iron deficiency has been considered as the most common cause of anemia in developing countries [18]. Our findings further support that iron deficiency is the most common cause for anemia in Nepalese children as indicated by poor iron status indicators in anemic children. Besides the lack of adequate iron in diet, other chronic conditions like hook worm infestation, malaria and genetic factors also contribute to high prevalence of anemia in developing countries [18].

Though, the median UIE (222 μg/L) in our study population was more than optimum, we found 19.8 % children had insufficient UIE, which is considered as iodine deficiency [19]. Our results suggest improvement on iodine status in Nepalese children. Higher median UIE than optimum as seen in our study also indicates excessive iodine intake and raises concern about the risk for iodine-induced hyperthyroidism [21]. The median UIE in the present study was slightly lower than in our previous study in Terai region where median UIE was 226.3 μg/L, but higher than in remote hilly regions where we reported median UIE of 187.52 μg/L [21, 22]. Since, soils in hills and mountains are reported to lack sufficient iodine and thus in the food derived from them, continuous provision of adequately iodized salt in such areas is necessary. Both, excess or insufficient intake of iodine adversely affects thyroid health, so optimum iodine intake should be maintained [23].

Present study reveals subclinical hypothyroidism (16.3 %) as the commonest thyroid dysfunction in Nepalese school children followed by overt hypothyroidism (1.3 %) and subclinical hyperthyroidism (1.8 %). In one community based study among school children in eastern Nepal, Chaudhari et al. reported that 31.8 % and 29.5 % children had subclinical hypothyroidism in Sunsari and Dhankuta districts respectively. However they did not reported any overt hypothyroidism or subclinical hyperthyroidism [24]. Study by Baral et al. at a hospital in eastern Nepal reported that 13.6 % population had hyperthyroidism and 17.1 % had hypothyroidism [13]. Previous studies in chronic disease patients like diabetes mellitus and chronic kidney diseases in eastern Nepal have reported thyroid dysfunction particularly subclinical hypothyroidism in large part of study patients [25, 26]. The high rate of hypothyroidism in the study subjects may be due to high rate of thyroid autoimmunity and deficiency of micronutrients like iron [25]. The most common cause of thyroid disorders worldwide is iodine deficiency, leading to goitre formation and hypothyroidism. In iodine-replete areas, most persons with thyroid disorders have autoimmune diseases like hashimoto’s thyroiditis and Graves’ disease [27]. The present study reveals no difference in thyroid function among iodine deficient and iodine sufficient children, and no association between UIE and thyroid hormones. Previous studies have also reported that there are usually no, or weak, associations between UIE and thyroid hormone concentrations [28].

In the present study, we observed significantly lower Hb level and iron status indicators in hypothyroids (overt and subclinical) than euthyroids, and iron deficiency and anemia in larger fraction of hypothyroid (both overt and subclinical) children. Our findings are in accord to results of previous studies by Bremner et al. and Banday et al. [29, 30]. Bremner et al. found serum iron concentrations significantly lower in participants with subclinical hypothyroidism than euthyroid subjects (*p* = 0.001), and Banday et al. reported iron deficiency in a significant portion of patients with primary hypothyroidism [29, 30]. It is believed that thyroid diseases affect the process of hematopoiesis and thyroid hormone deficiency may lead to bone marrow repression and/or decrease in erythropoietin production due to the reduction of O_2_ requirements. Thyroid hormones have also found to regulate the gene expression of transferrin [31].

Our findings suggest that anemic subjects tend to have higher TSH than non-anemic subjects, however, fT3 and fT4 does not seem to differ. Similarly, we observed that iron deficient children had significantly lower fT3 level than iron sufficient children. In a study by Metwalley et al., iron deficient children with hemoglobin level 10.9-7 gm/dl had significantly higher levels of serum fT3 and fT4 (*p* < 0.01 for both) and significantly lower levels of serum TSH (*p* < 0.05) as compared to patients with Hb level < 7 gm/dl [12]. In another study in Bangladesh among general population, serum TSH level was significantly higher (*p* < 0.05) and serum fT4 was significantly lower (*p* < 0.05) in iron deficient subjects than healthy controls, however, serum fT3 was almost similar between iron deficient and healthy controls [32]. Our finding demonstrates that anemic and iron deficient children have high risk for hypothyroidism, and hypothyroidism is associated with anemic and iron deficient children. Thus our findings suggests for the possibility that iron deficiency might impair thyroid metabolism as reported in previous studies. The two initial steps of thyroid hormone synthesis are catalyzed by heme containing enzyme, thyroid peroxidase. Severe iron deficiency may lower thyroid peroxidase activity and interfere with the synthesis of thyroid hormones thereby leading to hypothyroidism [33]. Studies have revealed that iron deficiency anemia (IDA) impairs thyroid metabolism and also decreases plasma total T4 and T3 concentrations and reduces peripheral conversion of T4 to T3 [9].

In the present study, we observed negative relation of transferrin saturation and Hb with TSH. Previous study by Bremner et al. reported significant relationships between free T3 and Hb, and inverse relationship of TSH with serum iron and transferrin saturation [29]. Bivolarska et al. also found slight positive statistically significant correlative association between the levels of free T4 and Hb (*r* = 0.217, *p* = 0.033) [11]. While many studies have found that both iron metabolism and thyroid functions are interdependent and each one of them could have a regulatory role, some studies have reported no association between thyroid hormones and anemia or iron deficiency [31–34]. In the study in Bangladesh, Akhter et al. observed no significant correlation of Hb concentration with serum TSH, fT3 and fT4 concentrations both in iron deficient and healthy control group [32]. Similarly, in the study in an area of mild iodine deficiency in Turkey, thyroid hormone levels of children with anemia were not significantly different from those without anemia, and there was no significant correlation between thyroid hormones and iron status [34]. There are several limitations of the present study. The sample size was small. Also, because of its cross-sectional nature we cannot draw conclusion about cause and effect relation between iron deficiency and thyroid dysfunction. Since, anti-thyroid peroxidase antibody (anti-TPO) was not assayed in the population, high prevalence of thyroid autoimmunity may also contribute for high rate of thyroid dysfunction in the study population.

## Conclusions

The study finds high prevalence of thyroid dysfunction, anemia and iron deficiency among Nepalese children and a significant association between iron status and thyroid function. Thus, anemia and iron deficiency seems to be associated with thyroid dysfunction particularly hypothyroidism. Future studies should be done in large samples and be directed toward finding the reasons for low thyroid hormones in anemic and iron deficient children.

## Abbreviations

Anti-TPO: Anti-thyroid peroxidase antibody
APDM: Ammonium persulphate digestion method
Free T3: Free triiodothyronine
Free T4: Free thyroxine
Hb: Hemoglobin
IDA: Iron deficiency anemia
TIBC: Total iron binding capacity
TSH: Thyroid stimulating hormone
UIE: Urinary iodine excretion
